# Predicting waist circumference from body mass index

**DOI:** 10.1186/1471-2288-12-115

**Published:** 2012-08-03

**Authors:** Samuel R Bozeman, David C Hoaglin, Tanya M Burton, Chris L Pashos, Rami H Ben-Joseph, Christopher S Hollenbeak

**Affiliations:** 1 Abt Associates Inc., Cambridge, MA, USA; 2 Independent consultant, Sudbury, MA, USA; 3 OptumInsight, Waltham, MA, USA; 4 United BioSource Corporation, Lexington, MA, USA; 5 Purdue Pharma L.P., Stamford, CT, USA; 6 Penn State College of Medicine, Department of Surgery, Hershey, PA, USA

## Abstract

**Background:**

Being overweight or obese increases risk for cardiometabolic disorders. Although both body mass index (BMI) and waist circumference (WC) measure the level of overweight and obesity, WC may be more important because of its closer relationship to total body fat. Because WC is typically not assessed in clinical practice, this study sought to develop and verify a model to predict WC from BMI and demographic data, and to use the predicted WC to assess cardiometabolic risk.

**Methods:**

Data were obtained from the Third National Health and Nutrition Examination Survey (NHANES) and the Atherosclerosis Risk in Communities Study (ARIC). We developed linear regression models for men and women using NHANES data, fitting waist circumference as a function of BMI. For validation, those regressions were applied to ARIC data, assigning a predicted WC to each individual. We used the predicted WC to assess abdominal obesity and cardiometabolic risk.

**Results:**

The model correctly classified 88.4% of NHANES subjects with respect to abdominal obesity. Median differences between actual and predicted WC were − 0.07 cm for men and 0.11 cm for women. In ARIC, the model closely estimated the observed WC (median difference: − 0.34 cm for men, +3.94 cm for women), correctly classifying 86.1% of ARIC subjects with respect to abdominal obesity and 91.5% to 99.5% as to cardiometabolic risk.

The model is generalizable to Caucasian and African-American adult populations because it was constructed from data on a large, population-based sample of men and women in the United States, and then validated in a population with a larger representation of African-Americans.

**Conclusions:**

The model accurately estimates WC and identifies cardiometabolic risk. It should be useful for health care practitioners and public health officials who wish to identify individuals and populations at risk for cardiometabolic disease when WC data are unavailable.

## Background

Body mass index (BMI), defined as weight in kilograms divided by the square of height in meters, has long been an important measure of excess body fat, and a high BMI is a well-recognized risk factor for cardiometabolic disorders. BMI is easily obtained and commonly assessed in clinical settings, and most current standards for overweight (BMI ≥ 25 kg/m^2^) and obesity (BMI ≥ 30 kg/m^2^) are based on BMI [1]. Current World Health Organization guidelines for cardiometabolic risk employ these thresholds [2]. Recent research, however, suggests that the distribution of body fat, for which BMI does not account, is a more important indicator of cardiovascular risk [3]. In particular, increased visceral fat has been shown to be an important risk factor for cardiovascular disease and diabetes, among other metabolic disorders [4-6]. An indicator of visceral fat, waist circumference (WC), is more sensitive to the distribution of body fat than is BMI [7], and therefore a better surrogate measure of android adiposity [8]. Previous work has attempted to define the link between the two measures by identifying BMI ranges that are equivalent in risk of cardiovascular events to WC ranges in current guidelines [9]. Current definitions of cardiometabolic risk factors, including guidelines issued by the National Cholesterol Education Program Adult Treatment Panel III (NCEP-ATP III) and International Diabetes Federation (IDF) for metabolic syndrome classification, include WC [10]. Previous research has demonstrated the prevalence of these conditions in well-characterized U.S. populations [11,12]. However, given the historical use of BMI in clinical practice, WC is not as widely available in established clinical databases. We sought to determine how closely WC could be predicted from BMI and two or three important demographic covariates — gender, age, and (optionally) race and ethnicity — as a basis for imputing WC when only BMI or weight and height are available, and to use the predicted WC to assess cardiometabolic risk.

## Methods

### Data

Following institutional review board approval by the Abt Associates Institutional Review Board, de-identified data were obtained for this study from two large studies: (1) the Third National Health and Nutrition Examination Survey (NHANES) 2001–2002 cohort and (2) the Atherosclerosis Risk in Communities Study (ARIC). The analytical model was built using data from NHANES, a national probability sample of the United States population collecting information on health and nutritional status [13]. Files contain both WC and BMI measurements, as well as demographic characteristics data, including age, gender, race, and ethnicity. The NHANES 2001–2002 data set contains data for 11,039 persons. We included individuals 18 years of age or older with no missing data on WC and BMI and excluded individuals with BMI > 40 kg/m^2^, which yielded a study sample of 4,641 adults.

To validate the gender-specific models, we applied them to data from ARIC, a prospective study of community sites in Maryland, Mississippi, Missouri, and North Carolina that examined risk factors and the natural history of atherosclerosis and cardiovascular disease in an ethnically diverse population [14]. A cohort of nearly 16,000 was examined at baseline (1987) and re-examined four times at 3-year intervals. Examinations included extensive laboratory tests and anthropometric assessments. In addition to periodic medical examinations, study staff conducted yearly follow-up telephone interviews to update the health status of each participant. Analyses presented here use data collected between 1996 and 1998 at Examination 4, which included 11,596 study participants.

### Model for WC

An initial model expressed the regression of WC on BMI in the following form:

(1) $$WC_{i}=b_{0}+b_{1}BMI_{i}+b_{2}AGE_{i}+b_{3}BLACK_{i}+b_{4}HISP_{i}+e_{i}$$

where *i* indexes individuals, *WC*_*i*_ is waist circumference for individual *i*, *BMI*_*i*_ is body mass index, *AGE*_*i*_ is current age (in years), *BLACK*_*i*_ is an indicator for African-American, *HISP*_*i*_ is an indicator for Hispanic ethnicity, and *e*_*i*_ is the residual. Because the relation of WC to BMI involved nonlinearity when BMI > 40 kg/m^2^ for both sexes, we based the models on data from individuals with BMI ≤ 40 kg/m^2^. (For individuals with BMI > 40 kg/m^2^ it is unlikely that a value of WC, either measured or predicted, will be needed as a guide to treatment.) An exploratory analysis, fitting a separate constant for each 2-year interval of age, revealed that the contribution of age to the model differed between men and women. For men the contribution was well summarized by a linear term in age, as in Equation 1. For women the pattern was better summarized by using one constant for age < 35 years and a separate intercept and slope for age ≥ 35 years. Thus, the model for women was

(2) $$WC_{i}=c_{0}+c_{1}BMI_{{}_{i}}+c_{{}_{2}}I\left\{AGE_{i}\geq 35\right\}+c_{3}AGE_{i}\times I\left\{AGE_{i}\geq 35\right\}+c_{4}BLACK_{{}_{i}}+c_{{}_{5}}HISP_{i}+e_{i}$$

where $I\left\{B\right\}$ is an indicator function: $I\left\{B\right\}=1$ when *B* is true and 0 otherwise. The contributions of race and ethnicity were adequately accounted for by those indicator variables; separate models were not necessary. Because NHANES used a complex sample design and oversampled certain segments of the population, the regressions took into account the sample design and survey weights.

We developed two additional models (one for men and one for women) omitting the *BLACK*_*i*_ and *HISP*_*i*_ variables. The results were essentially the same, and are available upon request.

### Validation

The linear regression models derived from the NHANES data yielded a predicted waist circumference for every individual in the ARIC dataset with BMI ≤ 40 kg/m^2^ and *AGE* < 70 years. We applied the restriction on *AGE* because the variable had been recoded to 70 for individuals who should have had *AGE* = 71, 72, or 73 years; the correct values were not available. In addition, we recalculated each individual’s BMI from the reported values of weight and height after we discovered that, for a small percentage of individuals, the reported BMI differed from the calculated BMI by a substantial amount. We computed the difference between observed and predicted waist circumference for each individual, and used the mean and various percentiles to summarize those differences. Examination of the distributions of differences from the two models revealed a moderate number of large values, which we investigated further.

Definitions of the seven cardiometabolic risk factor sets are presented in Table 1[10]. We assessed membership in each defined risk factor set using the actual and predicted waist circumference values for each individual. We computed sensitivity, specificity, and positive predictive value (PPV) using the predicted waist circumference as a “screening test” against the measured waist circumference as the “gold standard” for membership.

**Table 1:** Specifications of Cardiometabolic Risk Factor Sets

	**Metabolic Syndrome NCEP-ATP III**^**a**^	**Abdominal Obesity 1 (AO)**	**AO + Diabetes**	**AO + Dyslipidemia**	**AO + Diabetes + Dyslipidemia**	**Metabolic Syndrome IDF**^**a**^	**Abdominal Obesity 2**
	*≥3 of the following risk factors*	*High WC*	*High WC + diabetes*	*High WC + high TG or low HDL*	*High WC + diabetes + high TG or low HDL*	*High WC + 2 of the other 4 risk factors*	*High WC*
WC	Men ≥ 102 cm	Men ≥ 102 cm	Men ≥ 102 cm	Men ≥ 102 cm	Men ≥ 102 cm	Men ≥ 94 cm^b^	Men ≥ 94 cm^b^
	Women ≥ 88 cm	Women ≥ 88 cm	Women ≥ 88 cm	Women ≥ 88 cm	Women ≥ 88 cm	Women ≥ 80 cm^b^	Women ≥ 80 cm^b^
TG	≥ 150 mg/dL	NA	NA	≥ 150 mg/dL	≥ 150 mg/dL	≥ 150 mg/dL, or	NA
						on treatment for high TG	
HDL	Men < 40 mg/dL	NA	NA	Men < 40 mg/dL	Men < 40 mg/dL	Men < 40 mg/dL	NA
	Women < 50 mg/dL			Women < 50 mg/dL	Women < 50 mg/dL	Women < 50 mg/dL or	
						on treatment for low HDL	
BP	SBP ≥ 130 mm Hg or	NA	NA	NA	NA	SBP ≥ 130 mm Hg or	NA
	DBP ≥ 85 mm Hg or					DBP ≥ 85 mm Hg or	
	current use of anti-hypertensive medication					current use of anti-hypertensive medication	
						(Same as for NCEP)	
FPG	≥ 100 mg/dL or	NA	≥ 126 mg/dL or	NA	≥ 126 mg/dL or	≥ 100 mg/dL or	NA
	use of diabetes medication		use of diabetes medication		use of diabetes medication	previously diagnosed type 2 diabetes	NA

*WC* waist circumference, *TG* triglycerides, *HDL* high-density lipoprotein, *BP* blood pressure, *FPG* fasting plasma glucose, *NCEP* National Cholesterol Education Program, *ATP III* Adult Treatment Panel III.

^a^ Ford, E. S. Prevalence of the metabolic syndrome defined by the International Diabetes Federation among adults in the U.S. *Diabetes Care* 2005, 28:2745–2749.

^b^ For White and African American subjects. Refer to ^a^ Ford, E.S. article for other ethnic group-specific thresholds including for Mexican-American subjects.

## Results

Characteristics of persons in the NHANES 2001–2002 sample used to develop the model are representative of the U.S. population, with an average age near 44 years, predominantly white, and with an average BMI in the overweight but not obese range (Table 2). Coefficients for the models are presented in Table 3. To estimate WC, the coefficients and the values of the predictors are substituted into the equations. For example, the predicted WC of a 40-year-old white non-Hispanic man with a BMI of 35 kg/m^2^ is computed as: 22.61306 + 2.520738*(35) + 0.1583812*(40)− 3.703501*(0)− 1.736731*(0) = 117.2 cm. The predicted WC for a 50-year-old black non-Hispanic woman with a BMI of 28 kg/m^2^ is computed as 28.81919 + 2.218007*(28)− 3.688953*(1)+ 0.125975*(50)(1)− 0.6570163*(1) + 0.1818819*(0) = 92.9 cm.

**Table 2:** Characteristics of NHANES 2001–2 sample

	**Men(N = 2,246)**		**Women(N = 2,395)**	
	***Mean***	***Std. Error***	***Mean***	***Std. Error***
**Age**	43.95	0.559	44.64	0.531
**Race/Ethnicity**				
White	76.6%		76.1%	
Black	10.1%		11.1%	
Hispanic	13.3%		12.8%	
**Body Fat**				
WC	97.4	0.295	90.5	0.384
BMI	27.2	0.088	26.9	0.143
Overweight^a^	43.5%		30.3%	
Obese^b^	23.9%		28.2%	

^a^ BMI ≥ 25.

^b^ BMI ≥ 30.

**Table 3:** Models for predicting waist circumference from BMI

	**Men(N = 2,395)**			**Women(N = 2,246)**		
	***Coefficient***	***Std. Error***	***P-Value***	***Coefficient***	***Std. Error***	***P-Value***
Constant	22.61306	0.8792	< 0.0001	28.81919	0.8011	< 0.0001
Age	0.1583812	0.0049	< 0.0001	-	-	-
I {Age ≥ 35}	-	-	-	−3.688953	0.8308	< 0.0001
**Age×** I {Age ≥ 35}	-	-	-	0.125975	0.0118	< 0.0001
BMI	2.520738	0.0338	< 0.0001	2.218007	0.0318	< 0.0001
Black	−3.703501	0.3194	< 0.0001	−0.6570163	0.6730	0.344
Hispanic	−1.736731	0.4848	0.003	0.1818819	0.6729	0.79

**Figure 1:**
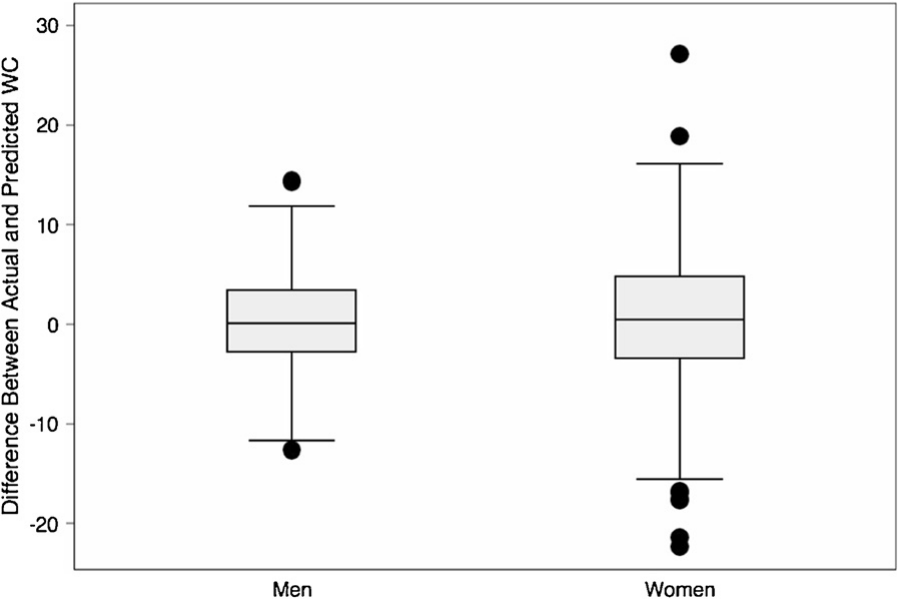
Boxplot of difference (cm) between actual and predicted WC (actual WC minus predicted WC) in NHANES participants age 54 to 69 years.

For men, the predicted WC is reasonably close to the actual WC, and for women it is somewhat less close than for men. The median difference (actual WC minus predicted WC) for men is − 0.07 cm, and the middle half of the differences extends from − 3.14 cm to + 3.00 cm. For women the median difference is 0.11 cm, and the quartiles are at − 3.84 cm and + 3.81 cm. To maximize comparability with ARIC, Figure 1 shows boxplots of the difference for men and women NHANES participants in the age range of 54 to 69 years. In this display the box covers the middle half of the data, from the 25^th^ percentile to the 75^th^ percentile, and the line across it shows the location of the median. Beyond lower and upper cutoffs (1.5 times the interquartile range below the 25^th^ percentile and above the 75^th^ percentile, respectively), data values are shown individually, and the “whiskers” indicate the range of the remaining data. For the 462 men in this age range, the median difference is − 0.15 cm, and the quartiles are at − 3.22 cm and + 3.21 cm. Three men had differences that were sizable enough to be shown at the ends of the boxplot (one negative and two positive). For the corresponding 458 women, the median difference is + 0.47 cm, and the quartiles are at − 3.66 cm and + 4.86 cm. Eight women’s differences are beyond the cutoffs (two high and six low). At the median the model for women underpredicts WC slightly, and the differences for women are considerably more variable than those for men.

Table 4 shows the characteristics of persons in the ARIC population used in validating the model. The average age of 61 is 17 years older than the NHANES population. (Participants in ARIC ranged in age from 45 to 64 years at Examination 1, so the range at Examination 4 would have been, with few exceptions, 54 to 73 years.) The ARIC participants contained more women than men (56.6% vs 43.4%), and more (especially women) were African-American. The population is predominantly white. The mean BMI and WC values are slightly higher for ARIC in men and women, with an average BMI in the overweight but not obese range.

**Table 4:** Characteristics of ARIC sample at Examination 4

	**Men (N = 3,806)**		**Women (N = 4,967)**	
	***Mean***	***Std. Dev***	***Mean***	***Std. Dev***
**Age (Years)**	61	4.5	61	4.5
**Race/Ethnicity**				
White	81%		75%	
Black	19%		25%	
Hispanic	0%		0%	
**Body Fat**				
WC (cm)	103	10.7	99	13.9
Derived BMI (kg/m^2^)	28.4	4.0	28.2	5.0
Height (cm)	176	6.5	162	5.9
Weight (lbs)	194	30.7	163	30.9
Overweight^a^	80%		71%	
Obese^b^	33%		35%	

^a^ BMI ≥ 25.

^b^ BMI ≥ 30.

**Figure 2:**
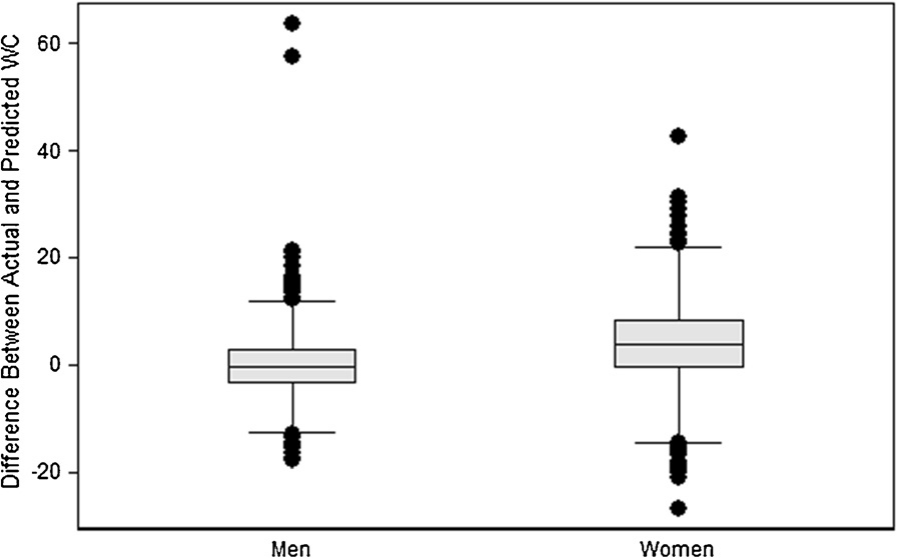
Boxplot of difference (cm) between actual and predicted WC (actual WC minus predicted WC) in ARIC participants.

As seen in Figure 2, for the ARIC study participants as a whole, the predicted WC is also reasonably close to the actual WC. For men the median difference is − 0.34 cm, and the quartiles are − 3.49 cm and + 2.74 cm. For women the median difference, + 3.94 cm, corresponds to underestimation of WC; the quartiles, − 0.79 cm and + 8.47 cm, yield an interquartile range of 9.26 cm, roughly 1.5 times the interquartile range for men. Of the 3,806 men, 33 had differences that were outside the cutoffs of the boxplot (12 negative and 21 positive). Only the most extreme of these, however, would normally be regarded as outliers. The largest two differences, 63.6 and 57.4, were caused by errors in the ARIC data, as we discovered from relating their height, weight, and BMI on Examination 4 to their height and weight on the previous examinations. Of the 4,967 women, 37 had differences beyond the cutoffs (23 negative and 14 positive). The largest positive and negative differences reflected problems in the data. Aside from participants with apparently misrecorded data, those with extreme differences seemed to have combinations of height, weight, and WC that simply were not well fitted by the models. We also noticed that, of the 37 women with extreme residuals, 20 were black non-Hispanic. The similarity between Figure 2 and Figure 1 indicates that, in the absence of problems in the data, the models performed nearly as well in predicting WC for individuals in ARIC as they did for individuals in NHANES.

The difference between actual and predicted WC, however, is not uniform over the range of WC. In both NHANES and ARIC and for both men and women, the models tend to overpredict when WC is small and underpredict when WC is large. Among men in both databases overprediction is noticeable (median 2 to 3 cm in WC intervals of width 5 cm) for WC < 95 cm. A similar degree of underprediction occurs for men in NHANES with WC ≥ 115 cm and for men in ARIC with WC ≥ 125 cm. Among women in both databases the pattern was substantially stronger: overprediction of 3 to 5 cm (median) for WC < 85 cm, underprediction of around 5 to 7 cm (median) for WC ≥ 115 cm in NHANES, and underprediction increasing steadily in ARIC from 3 cm (median) for 90 cm ≤ WC ≤ 94 cm to 9 cm (median) for 120 cm ≤ WC ≤ 124 cm. These patterns affect membership in the risk factor sets, discussed below.

Tables 5 and 6 present the results of using the predicted WC value to define membership in the seven cardiometabolic risk factor sets for women and men, respectively. For every risk factor set, the WC prediction model is slightly more successful (i.e., a higher positive predictive value and a higher proportion correctly identified as belonging to a particular risk factor set) for women than for men. For women, the proportion correctly identified using the predicted WC was 93.2% or higher for all sets except for the higher abdominal obesity threshold (≥ 88 cm) alone, where the proportion correctly identified was 86.8%. The misclassified individuals (13%) were twice as likely to be false negatives (i.e., incorrectly classified as not obese) as opposed to false positives. For this risk factor set, both specificity (correctly identifying non-membership for those who are non-members — 82.6%) and sensitivity (correctly identifying membership for those who are members — 88.0%) were low. For the risk factor sets excluding metabolic syndrome, the proportion correctly identified increases as the number of defining criteria increases, with 99.5% correctly identified for the abdominal obesity plus diabetes plus dyslipidemia set.

**Table 5:** Risk Factor Set Membership from Predicted WC in ARIC Women; BMI ≤ 40; Age < 70. (N = 4967)

**Risk Factor Set**	**Observed proportion (N) in risk set**	**Positive Predictive Value**	**Sensitivity**	**Specificity**	**Proportion correctly predicted**
NCEP-ATP III Metabolic Syndrome	50.2% (2495)	98.1%	95.4%	98.2%	96.8%
IDF Metabolic Syndrome	55.2% (2744)	98.7%	98.7%	98.3%	98.5%
Abdominal Obesity1 (AO1)	76.4% (3795)	94.2%	88.0%	82.6%	86.8%
AO1 and DM	13.6% (675)	98.8%	95.3%	99.8%	99.2%
AO1 and Dyslipidemia	45.2% (2243)	96.9%	90.7%	97.6%	94.5%
AO1, DM and Dyslipidemia	9.6% (479)	98.9%	96.0%	99.9%	99.5%
Abdominal Obesity 2	91.8% (4560)	95.7%	97.0%	51.4%	93.2%

*NCEP-ATP III* National Cholesterol Education Program, Adult Treatment Panel III.

*IDF* International Diabetes Foundation.

Abdominal Obesity 1: ≥ 88 cm.

Abdominal Obesity 2: ≥ 80 cm.

*DM* Diabetes Mellitus.

**Table 6:** Risk Factor Set Membership from Predicted WC in ARIC Men; BMI ≤ 40; Age < 70. (N = 3806)

**Risk Factor Set**	**Observed proportion (N) in risk set**	**Positive Predictive Value**	**Sensitivity**	**Specificity**	**Proportion correctly predicted**
NCEP-ATP III Metabolic Syndrome	49.8% (1895)	94.5%	96.9%	94.5%	95.7%
IDF Metabolic Syndrome	58.9% (2242)	93.8%	94.1%	91.1%	92.9%
Abdominal Obesity 1 (AO1)	48.8% (1857)	83.9%	87.4%	84.0%	85.7%
AO1 and DM	11.9% (454)	90.3%	89.9%	98.7%	97.6%
AO1 and Dyslipidemia	33.3% (1266)	86.0%	89.0%	92.8%	91.5%
AO1, DM and Dyslipidemia	9.2% (352)	90.3%	89.8%	99.0%	98.2%
Abdominal Obesity 2	81.2% (3089)	92.8%	93.0%	68.9%	88.5%

*NCEP-ATP III* National Cholesterol Education Program, Adult Treatment Panel III.

*IDF* International Diabetes Foundation.

Abdominal Obesity 1: ≥ 102 cm.

Abdominal Obesity 2: ≥ 94 cm.

*DM* Diabetes Mellitus.

The positive predictive value (PPV) results using the predicted WC were 94.2% or higher for each of the risk factor sets. The lowest PPV was for abdominal obesity as defined by the 88 cm threshold alone (94.2%), and the highest PPVs were observed for the metabolic syndrome definitions, abdominal obesity (AO) plus diabetes, and AO plus dyslipidemia plus diabetes (all above 98%). Sensitivity using predicted WC was higher than specificity for the abdominal obesity boundaries alone and for the IDF-defined metabolic syndrome. Specificity was higher for NCEP-defined metabolic syndrome, AO plus diabetes, AO plus dyslipidemia, and AO plus diabetes plus dyslipidemia. Except for the abdominal obesity alone definitions, both sensitivity and specificity were 90.7% or higher for every risk factor set. Specificity was low (51.4%) for the 80 cm abdominal obesity threshold, indicating a high number of false-positive predictions, and thus reflecting the systematic overprediction of WC among thinner women discussed above.

For men (Table 6), the proportion correctly identified as belonging to a risk factor set using the predicted WC was 91.5% or higher for all sets except abdominal obesity alone. For the higher criterion of greater than or equal to 102 cm, the proportion correctly identified was 85.7%; for the lower criterion of greater than or equal to 94 cm, the proportion correctly identified was 88.5%. Particularly for the lower (94 cm) threshold, the misclassified individuals were more likely to be false positives (i.e., incorrectly classified as obese) than false negatives, as indicated by specificities lower than sensitivities, and reflecting the systematic overprediction of WC among thinner men discussed above. For the risk factor sets excluding metabolic syndrome, the proportion correctly identified increased as the number of defining criteria increased, with 98.2% correctly identified for the abdominal obesity plus diabetes plus dyslipidemia set.

The positive predictive value (PPV) results using the predicted WC for men were lowest for those risk factor sets using the higher 102 cm threshold, ranging from 83.9% for AO alone to 90.3% for AO plus diabetes plus dyslipidemia. The highest PPVs, 93.8% and 94.5%, were observed for the IDF and NCEP metabolic syndrome definitions, respectively. Sensitivity using predicted WC was higher than specificity for the metabolic syndrome risk factor sets and for the AO alone sets.

We also compared the results of the waist circumference prediction model to an approach using BMI threshold values to predict risk factor set membership, using the WHO-recommended BMI values to define membership in the six cardiometabolic risk factor sets. Specifically, we substituted the WHO-recommended BMI values for the waist circumference criteria as follows: BMI ≥ 25 kg/m^2^ (“overweight”) corresponds to WC of 80 cm for women and 94 cm for men, and BMI ≥ 30 kg/m^2^ (“obese”) corresponds to WC of 88 cm for women and 102 cm for men. Among ARIC women, the WC prediction model categorized risk factor set membership more accurately than the BMI threshold for every set. In contrast, among ARIC men the results of the two approaches differed much less. The differences in the proportions correctly identified were within one percentage point for three of the seven sets, and the WC prediction model categorized risk factor set membership more accurately than the BMI threshold in the other four sets. Tables presenting these results are available upon request.

We repeated all of the analyses described above for a simpler model omitting the race and ethnicity demographic variables. The results were essentially the same. Analogous Figures 1 and 2 (the boxplots showing the predicted WC values) and Tables 5 and 6 (the validation exercise assessing the sensitivity, specificity, positive predictive value, and proportion correctly predicted) are available upon request.

## Discussion

We constructed a model estimating WC from BMI and three demographic variables — gender, age, and race/ethnicity — because WC is increasingly recognized as a more accurate and therefore more useful metric than BMI alone for predicting cardiometabolic risk. Using the estimated WC value from the model more accurately predicted cardiometabolic risk than using BMI alone. Because the predictor variables are generally collected in clinical practice and therefore available for assessment by practitioners as well as health plans and public health officials, this model may assist in the accurate identification of individuals at higher risk for cardiometabolic events. This would be the result even if WC is not measured and actual WC measurements are not available. As well, an alternative model is available for practitioners and researchers to whom data on race or ethnicity are not readily available.

The model is generalizable to Caucasian and African-American adult populations because it was constructed from data in a large, population-based sample of U.S. men and women, and then validated in a population with a larger representation of African-Americans. This model has not been validated in non-Caucasian, non-African-American ethnic groups. Further work in that regard should be conducted.

Although the model successfully estimates WC and, using the WC estimate, membership in the risk clusters, we observed a modest level of systematic error in the model results. Specifically, the model tends to overestimate the true WC in lower ranges of WC, and underestimate the true WC in upper ranges of WC, particularly among women. Heterogeneity in the anthropometry of women (e.g., obesity in hips, thighs, and mammary tissue not measured by WC) compared with men likely accounts for the greater mean differences in the predicted and observed WC values for women as compared with men. Efforts to identify a correction term in the model for the NHANES data were unsuccessful. However, the magnitude of the systematic error is sufficiently small that using the estimated WC to identify cardiometabolic risk is accurate in both genders, and slightly better for women.

## Conclusions

The model accurately estimates WC and identifies cardiometabolic risk. It should be useful for health care practitioners and public health officials who wish to identify individuals and populations at risk for cardiometabolic disease when WC data are unavailable.

## Competing interests

The majority of the work overall, including this manuscript, has not been performed with sponsorship funding. Initial aspects of this work were sponsored by Sanofi-Aventis, which funded Abt Bio-Pharma Solutions, a subsidiary of Abt Associates, Inc., to lead this study. In that initial stage, SRB performed the research as an employee of Abt Associates; DCH, TMB and CLP performed this study as employees of Abt Bio-Pharma Solutions, now part of United BioSource Corporation; RHBJ contributed as an employee of Sanofi-Aventis; and CSH contributed as an employee of Pennsylvania State University.

## Authors’ contributions

All authors made substantive intellectual contributions to this study. SRB and DCH contributed to study design and analysis; drafted the initial version of the manuscript; and contributed to its critical revision. SRB, TMB and CLP contributed to data acquisition. TMB, CLP, RHBJ and CSH also contributed to the study design, analysis, and critical revision of the manuscript for important intellectual content. Each author has participated sufficiently in the work to take public responsibility for manuscript content, and has read and approved the final manuscript.

## Pre-publication history

The pre-publication history for this paper can be accessed here:

http://www.biomedcentral.com/1471-2288/12/115/prepub
